# *In vitro* activity of aztreonam–avibactam combination against blood culture isolates of *Stenotrophomonas maltophilia* in Japan before the launch of ceftazidime–avibactam

**DOI:** 10.1128/spectrum.03316-24

**Published:** 2025-03-14

**Authors:** Wataru Aoki, Yoshifumi Uwamino, Yuka Kamoshita, Rika Inose, Mika Nagata, Naoki Hasegawa, Hiromichi Matsushita

**Affiliations:** 1 Clinical Laboratory, Keio University Hospital 34787 , Tokyo, Japan; 2 Department of Laboratory Medicine, Keio University School of Medicine, Tokyo, Japan; 3 Department of Infectious Diseases, Keio University School of Medicine, Tokyo, Japan; Seton Hall University, South Orange, New Jersey, USA

**Keywords:** aztreonam, avibactam, *Stenotrophomonas maltophilia*, blood culture, ceftazidime–avibactam

## LETTER

*Stenotrophomonas maltophilia* is an environmental organism that causes severe infections in patients with underlying diseases. The treatment options are limited. Current guidelines list aztreonam (AZT) in combination with ceftazidime (CAZ)–avibactam (AVI) and cefiderocol as the preferred treatment option (1). This combination is effective because AZT is not a substrate for L1 β-lactamase, whereas AVI exhibits inhibitory activity against L2 β-lactamase (2).

AVI, marketed as CAZ–AVI, is already in use in more than 90 countries (3). Susceptibility data for the AZT–AVI combination have been reported (4, 5), predominantly originating from countries in which CAZ–AVI is already in use. The data suggest that local usage may influence susceptibility patterns. In Japan, CAZ–AVI (Zavicefta) was launched in November 2024.

This study aimed to evaluate the *in vitro* activity of AZT–AVI against *S. maltophilia* isolates obtained from blood cultures prior to the introduction of CAZ–AVI in Japan. The study included the preserved first clinical isolates of *S. maltophilia* from 53 patients from whom *S. maltophilia* was recovered from blood cultures at Keio University Hospital (Tokyo, Japan) between January 2012 and October 2024. The median patient age was 66 years, with 35 male and 18 female patients. Infection foci included catheter-related blood stream infection (*n* = 24), surgical site (*n* = 6), hepatobiliary (*n* = 5), pneumonia (*n* = 3), and unknown (*n* = 15). Strain identification was confirmed using a MALDI Biotyper. The minimum inhibitory concentrations (MICs) for AZT, AZT–AVI, CAZ, and CAZ–AVI were determined using broth microdilution of cation-adjusted Mueller–Hinton broth according to the Clinical and Laboratory Standards Institute guidelines (6). Bacterial suspensions were adjusted to a McFarland standard of 0.5, with AZT and CAZ tested across a concentration range of 0.063–128.0 µg/mL, with AVI included at a fixed concentration of 4 µg/mL. Plates were incubated aerobically at 35°C for 20 h, and the MICs were recorded. Quality control was performed using *Klebsiella pneumoniae* ATCC 700603.

**Fig 1:**
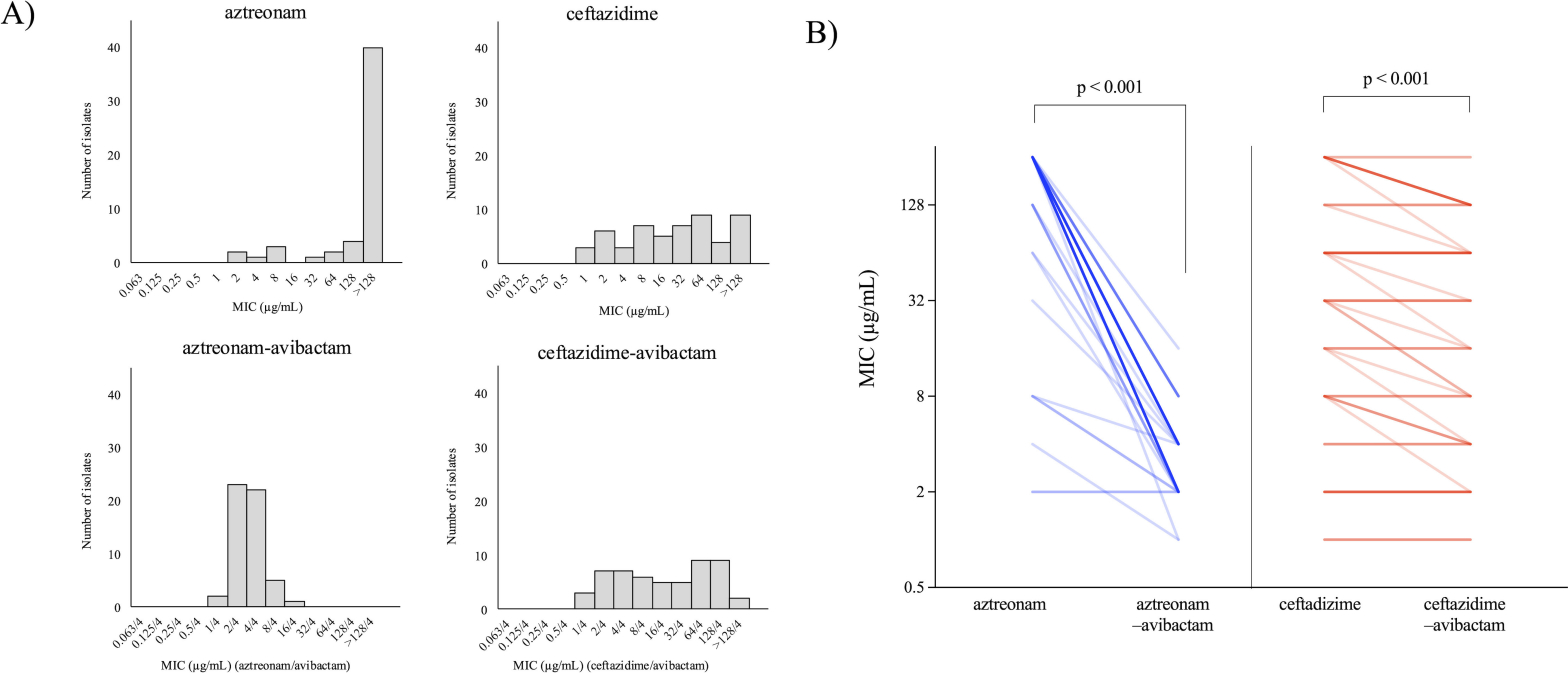
Distribution of MICs for aztreonam, aztreonam–avibactam, ceftazidime, and ceftazidime–avibactam and effect of avibactam combination on MIC reduction. Panel A shows the distribution of isolates of S. maltophilia obtained from blood cultures based on MIC values measured by the broth microdilution method. For MICs of drug combinations with avibactam, the concentration of aztreonam or ceftazidime is indicated first, followed by the concentration of avibactam. Panel B shows the *in vitro* reduction in MICs of aztreonam and ceftazidime after the addition of 4 µg/mL avibactam. The line intensity varies in proportion to the number of overlapping isolates. Statistical analysis was performed using the logarithm of MICs with a base of 2, and the Wilcoxon signed-rank test was used to assess the statistical significance of the changes. Statistical analyses were conducted using JMP Pro 17.

Both the MIC_50_ and the MIC_90_ for AZT alone were >128 µg/mL, whereas those for AZT–AVI were 4/4 µg/mL and 8/4 µg/mL, respectively. For CAZ and CAZ–AVI, the MIC_50_ values were 32 and 16/4 µg/mL, respectively, and the MIC_90_ values were >128 and 128/4 µg/mL, respectively (Fig. 1A). Both AZT and CAZ showed a reduced MIC when combined with AVI; however, the reduction was more pronounced with AZT (Fig. 1B).

The *in vitro* activity of AZT–AVI against *S. maltophilia* blood culture isolates was comparable to that of reports from regions where CAZ–AVI was already in use (4, 5, 7), despite it not being in use in Japan during the study period. Although clinical breakpoints for AZT–AVI have not been established, our findings suggest that this combination could be used for the treatment of *S. maltophilia* bacteremia in Japan. Although this study did not directly compare AZT–AVI susceptibility before and after the introduction of CAZ–AVI in Japan, the data suggest that the *in vitro* activity of AZT–AVI may not be substantially affected by regional variations in CAZ–AVI usage. Future studies are needed to monitor possible susceptibility changes as CAZ–AVI use increases.
